# The cross-sectional and longitudinal relationship between overgeneral autobiographical memory and adolescent depression in a UK population-based cohort

**DOI:** 10.1016/j.jad.2020.02.011

**Published:** 2020-04-01

**Authors:** Warne N, Caseras X, Rice F

**Affiliations:** a MRC Centre for Neuropsychiatric Genetics and Genomics, Division of Psychological Medicine and Clinical Neurosciences, Cardiff University, Cardiff, United Kingdom; b Population Health Sciences, Bristol Medical School, University of Bristol, Bristol, United Kingdom

**Keywords:** Overgeneral autobiographical memory, Depression, Longitudinal, ALSPAC, OGM, overgeneral autobiographical memory, ALSPAC, Avon Longitudinal Study of Parents and Children, AMT, Autobiographical Memory Test

## Abstract

**Background:**

Overgeneral autobiographical memory (OGM), the tendency to recall fewer specific memories and recall more repeated or extended events, is associated with subsequent adult depression. However, prospective associations are only found in adolescents with additional risk factors for depression (e.g. OGM for negative material is associated with subsequent depression in females and those at familial risk of depression) and not in community samples. It remains unclear whether OGM is associated with subsequent depression in population-based adolescent samples or just in high-risk adolescents.

**Methods:**

We examined the relationship between OGM for negative cues (age 13) and adolescent depressive symptoms in a population-based cohort - the Avon Longitudinal Study of Parents and Children. Regression models investigated the association of OGM for negative cues with depressive symptoms at age 12.5 years (*n* = 3,145) and age 16.5 years (*n* = 2,345). Associations with alternative measures of OGM were also explored. Gender and maternal depression were examined as potential moderators of these relationships.

**Results:**

OGM for negative cues was associated with both contemporaneous and prospective depressive symptoms. Only OGM for negative cues and total OGM were prospectively associated with depressive symptoms. There was no evidence of moderation by gender or maternal depression.

**Limitations:**

Depression was reported 6 months earlier than OGM.

**Conclusions:**

To our knowledge, this is the first study to find a prospective link between OGM for negative material and depression in a population-based cohort. Results highlight memory biases can precede subsequent adolescent depression in the general population, not just high-risk samples.

## Highlights

• Negative overgeneral memory predicted subsequent adolescent depressive symptoms.
• This association was present in the whole sample, not just high-risk adolescents.
• This prospective link is novel in samples without increased risk for depression.
• Overgeneral memory may be a risk factor for adolescent depressive symptoms.
• Overgeneral memory definition and valence are important to consider in future work.

## Introduction

A number of cognitive biases in self-referential processing, attention and memory, and in the cognitive control of emotional information are evident in depression, with some of the most robust evidence for memory biases (Gotlib and Joormann, 2010). Depressed individuals remember their personal past differently from non-depressed individuals (Gotlib and Joormann, 2010): when asked to recall from memory an event that occurred at a particular time and place (i.e. a specific autobiographical memory), depressed adults and adolescents instead recall more overgeneral autobiographical memories that cover extended time periods or repeated events. This phenomenon is known as overgeneral autobiographical memory (OGM) (Hitchcock et al., 2014; Williams et al., 2007). Recalling more general autobiographical memories rather than specific instances has similarities to the overgeneral and abstract processing that has been reported in depression more broadly including the cognitive bias of drawing general conclusions from specific instances (Beck, 2008; Watkins, 2004). Other memory biases associated with depression include mood congruent recall whereby depressed individuals recall more memories for negative material and recall such memories more quickly (Gotlib and Joormann, 2010; Timbremont and Braet, 2004). One theory of OGM suggests that this phenomenon develops first for negative memories but can then become generalised (Williams et al., 2007). OGM affects a number of processes that are disrupted in depression such as mood regulation, social problem solving, sense of self and self-esteem (Gotlib and Joormann, 2010; Williams et al., 2007). Thus, being unable to recall specific personal memories could promote difficulties in adaptive emotion processing, relationships with others and self-reflection, which may lead to mood disturbances over time.

Identifying whether OGM is prospectively associated with depression is important as temporal precedence indicates OGM may be a risk factor involved in the development of depression. Identified causal risk factors could be potential clinical targets for interventions before the first episode of depression. Current evidence suggests that OGM increases the risk of subsequent depression in adults (Sumner et al., 2010). However, evidence of this prospective link in youth samples is inconsistent and may differ according to the type of sample. Studies using adolescent samples at increased risk of depression (including females with high depressive symptoms, offspring of depressed parents, and those with prior depressive episodes) have reported prospective associations (Hipwell et al., 2011; Rawal and Rice, 2012; Sumner et al., 2011) while studies using community samples failed to report associations (Crane et al., 2016; Gutenbrunner et al., 2017), except in the presence of additional risk factors (Hamlat et al., 2015; Stange et al., 2013). These results have led to suggestions that a prospective relationship between OGM and depression may exist only in samples where additional depression vulnerability factors are present (Crane et al., 2016; Gutenbrunner et al., 2017). Alternatively, methodological differences between studies may explain inconsistent findings. In particular, the definition and valence of OGM and gender may be important to consider. Some studies report associations only for OGM elicited by negative cues (Rawal and Rice, 2012); there is strong evidence of mood congruent recall (Gotlib and Joormann, 2010) and theoretical suggestions that OGM develops first for negative material (Williams et al., 2007). Furthermore, several studies report prospective associations between OGM and adolescent depression only for females (Hamlat et al., 2015; Hipwell et al., 2011; Rawal and Rice, 2012).

We explored the association between OGM and adolescent depression in a large population-based sample and asked:

1 Is the association between OGM and adolescent depressive symptoms stronger in a group at elevated risk for depression (the offspring of depressed parents)?
2 Is the prospective association between OGM and depression greater for OGM to negative material than other measures of OGM (e.g. for positive material)?
3 Are these relationships moderated by gender?

In a single sample, we therefore aimed to test the longitudinal relationship between OGM and adolescent depression symptoms and to examine potential reasons for discrepant findings according to sample type (elevated risk for depression versus community sample) and alternative measures of OGM (negative versus positive material).

## Methods

### Sample

Data were from a prospective, longitudinal birth cohort - the Avon Longitudinal Study of Parents and Children (ALSPAC) (Boyd et al., 2013; Fraser et al., 2013). The core sample consisted of 14,541 pregnant mothers who were expecting to deliver between 1st April 1991 and 31st December 1992 in Avon, England. Of these pregnancies, 13,988 children were alive at 1 year. The sample was supplemented when the oldest children were 7 years old with participants who failed to join the study at the initial recruitment stage (*n* = 913, total *n* = 14,901). The study website contains details of all available the data that is available through a fully searchable data dictionary and variables search tool: http://www.bristol.ac.uk/alspac/researchers/our-data/. There were 4,111 individuals with data on autobiographical memory (age 13) and on maternal depression. Of these, 3,154 individuals (55% female) also had depression data at age 12.5 years and 2,345 individuals (59% female) had depression data at age 16.5 years.

### Procedure

Ethical approval was obtained from the ALSPAC Ethics and Law Committee and Local Research Committees. Data were collected at various time points throughout pregnancy and after birth via questionnaires and face-to-face clinics. Project approval was gained from the ALSPAC Executive Committee (project number b2647).

### Measures

**Autobiographical memory:** Adolescents completed a written Autobiographical Memory Test (AMT) at age 13 (Crane et al., 2016; Heron et al., 2012). Participants were instructed to write a real memory for 10 words (5 positive: e.g. ‘happy’; 5 negative: e.g. ‘failure’) or leave the space blank if they could not recall a memory. Each response was coded for specificity into one of five categories (specific, extended, categoric, semantic associate, omission - see Supplementary Material). We examined specific and overgeneral memory (Williams et al., 2007). Specific responses were single events occurring on one day, at a particular time and place; overgeneral responses combined extended (events lasting more than two days) and categoric responses (repeated events). The primary predictor variable was number of overgeneral memories to negative cues (OGMneg). Secondary variables were total number of overgeneral memories (OGMtotal), overgeneral memories to positive cues (OGMpos), total specific memories (AMStotal), specific memories to negative cues (AMSneg) and specific memories to positive cues (AMSpos).

**Maternal depression:** A variable of severe, recurrent maternal depression was derived from maternal reports on questionnaires from study child pregnancy to age 12. Mothers were coded as having depression if they reported at least two separate instances of depression with one instance being severe (Hammerton et al., 2013).

**Adolescent depressive symptoms:** were assessed by self-reports on the 13-item version of the Mood and Feelings Questionnaire (sMFQ; Angold et al., 1995) at 12.5 and 16.5 years. Parent reports on the sMFQ at 13 and 16.5 years were used in sensitivity analyses.

**Covariates:** Analyses adjusted for age, gender and IQ as they have been associated with OGM (Heron et al., 2012; Williams et al., 2007). IQ was assessed using the WISC-III (Wechsler, 1991) at age 8. Indicators of socioeconomic status (SES) used in sensitivity analyses were: 1) parity (first, second, or third (or more) born) via maternal report at 18 weeks gestation; 2) economic disadvantage (coded ‘yes’ (1); ‘no’ (0) as per the international definition of poverty ≤60% of the median income of the sample (Gordon, 2006) at 11 years); 3) maternal highest educational qualifications reported at 32 weeks gestation (‘A levels/degree’ (1); ‘lower than A levels’ (0)).

### Statistical analysis

Analyses were performed in Stata version 13. Associations between OGM and depressive symptoms were examined using linear regression with standardised predictors. Covariates (IQ, gender (male (0), female (1)), age, maternal depression status; plus baseline depressive symptoms for prospective analyses) were included in Step 1. The main effect of OGMneg was included in Step 2. Moderation by maternal depression status was examined by including the interaction between OGMneg and maternal depression in Step 3. Moderation by gender was examined in Step 3. Where there was a significant main effect of OGMneg, analyses were repeated using secondary indicators of autobiographical memory (OGMtotal, OGMpos, AMStotal, AMSneg, AMSpos) to assess the role of memory definition and valence.

Sensitivity analyses were performed repeating OGMneg analyses with parent-reported adolescent depressive symptoms to test if results were robust to informant. Additional sensitivity checks adjusted for economic disadvantage, parity and maternal education. Multiple imputation by chained equations was used to examine the impact of missing data on results (full details in Supplementary Material).

## Results

Table 1 shows descriptives and correlations for key study variables. OGMneg was correlated with depressive symptoms at baseline (*r* = 0.112, *p* < 0.001) and follow-up (*r* = 0.116, *p* < 0.001).

**Table 1:** Descriptive information and correlations between autobiographical memory, depression and covariates.

	1	2	3	4	5	6	7	8	9	10	11	12	13
1. Gender (female)													
2. Age	**−0.099**												
3. IQ	−0.020	**−0.048**											
4. Economic disadvantage	0.014	0.009	**−0.143**										
5. Maternal depression status	**0.046**	0.022	**−0.052**	**0.215**									
6. Depressive symptoms age 12.5	**0.121**	0.004	−0.010	0.020	**0.081**								
7. Depressive symptoms age 16.5	**0.225**	−0.007	**−0.043**	**0.068**	**0.129**	**0.337**							
8. OGMtotal	−0.014	−0.018	**−0.106**	−0.004	−0.012	**0.065**	**0.079**						
9. OGMneg	**0.046**	**−0.033**	**−0.072**	0.011	0.001	**0.112**	**0.116**	**0.864**					
10. OGMpos	**−0.071**	0.002	**−0.110**	−0.019	−0.021	−0.002	0.019	**0.858**	**0.483**				
11. AMStotal	**0.149**	−0.022	**0.166**	**−0.042**	−0.005	**0.065**	**0.051**	**−0.343**	**−0.185**	**−0.409**			
12. AMSneg	**0.118**	−0.012	**0.149**	**−0.045**	−0.011	**0.105**	**0.061**	**−0.266**	**−0.248**	**−0.210**	**0.874**		
13. AMSpos	**0.145**	−0.027	**0.146**	−0.030	<0.001	0.015	0.032	**−0.339**	**−0.087**	**−0.501**	**0.898**	**0.572**	
Mean or %	54.90%	157.337	107.235	16.60%	18.44%	3.965	5.789	2.564	1.319	1.244	3.787	1.359	2.427
SD or n	2257/4111	1.193	15.820	582/3505	758/4111	3.791	5.547	2.121	1.244	1.220	2.499	1.340	1.478

AMSneg – specific autobiographical memories to negative cues; AMSpos - specific autobiographical memories to positive cues; AMStotal – total specific autobiographical memories; OGMneg – overgeneral autobiographical memories to negative cues; OGMpos – overgeneral autobiographical memories to positive cues; OGMtotal – total overgeneral autobiographical memories. Correlations significant at *p* < 0.05 indicated in bold.

Table 2 shows results of regression analyses examining the association between OGMneg and depressive symptoms. OGMneg was associated with greater contemporaneous and prospective depressive symptoms after adjusting for covariates. No moderation by maternal depression status (Table 2) or gender (Supplementary Table 4) was observed.

**Table 2:** Regression models with main effect of OGMneg predicting contemporaneous and prospective depressive symptoms and maternal depression interactions.

	Contemporaneous depressive symptoms					Prospective depressive symptoms				
	(*n* = 3,154)					(*n* = 2,345)				
	Model change			Coefficients		Model change			Coefficients	
	R^2^	p	β	B (95% CI)	p	R^2^	p	β	B (95% CI)	p
**Step 1: Covariates**	0.018	<0.001				0.158	<0.001			
Full scale IQ			−0.005	−0.020 (−0.158, 0.117)	0.773			−0.024	−0.139 (−0.355, 0.078)	0.209
Age			0.015	0.075 (−0.103, 0.254)	0.407			−0.007	−0.068 (−0.438, 0.303)	0.720
Gender			0.116	0.878 (0.615, 1.140)	<0.001			0.195	2.153 (1.738, 2.568)	<0.001
Maternal depression			0.066	0.679 (0.324, 1.034)	<0.001			0.072	1.106 (0.529, 1.683)	<0.001
Age 12.5 depressive symptoms								0.309	1.779 (1.562, 1.996)	<0.001
**Step 2: Main effect**	Δ0.011	<0.001				Δ0.005	<0.001			
OGMneg			0.107	0.406 (0.275, 0.537)	<0.001			0.074	0.410 (0.202, 0.617)	<0.001
**Step 3: Moderation by maternal depression**	Δ<0.001	0.388				Δ<0.001	0.791			
OGMneg x Maternal Depression Status			0.017	0.159 (−0.202, 0.519)	0.388			−0.005	−0.080 (−0.675, 0.514)	0.791

OGMneg – overgeneral autobiographical memory to negative cues. Results significant at *p* < 0.05 are indicated in bold.

For alternative indicators of autobiographical memory, there were main effects of total OGM, AMStotal and AMSneg such that *more* memories were associated with *more* contemporaneous depressive symptoms (Supplementary Table 4). Maternal depression status moderated relationships between AMStotal, AMSneg and AMSpos and contemporaneous depressive symptoms, with stronger associations for participants with a depressed mother (Supplementary Table 4). The AMSneg by gender interaction was also associated with contemporaneous depressive symptoms with stronger effects for females (Supplementary Table 4).

Total number of OGMs, but no other alternative memory indicator, was associated with subsequent depressive symptoms prospectively (Supplementary Table 4).

Sensitivity analyses showed the same pattern of results was observed when using parent-reported depressive symptoms as the outcome measure and additionally adjusting for SES (Supplementary Table 4). The relationships between OGM and depression using imputed data were consistent with complete case analysis (Supplementary Table 4).

## Discussion

Overgeneral autobiographical memory (OGM) for negative cues was associated with adolescent depressive symptoms both contemporaneously and at 3-year follow-up in a large population-based cohort. This relationship was not altered by gender or maternal depression status.

Other indicators of autobiographical memory were associated with contemporaneous depressive symptoms in that more total specific memories and more specific memories for negative cues were associated with *more* contemporaneous depressive symptoms. The direction of this effect was unexpected given that depression has been associated with reductions in memory specificity (Williams et al., 2007), but could be explained by mood congruence bias, whereby adolescents with more depressive symptoms may be more likely to recall more detailed negative memories (Gotlib and Joormann, 2010; Timbremont and Braet, 2004). The only alternative indicator of autobiographical memory associated with prospective depressive symptoms was total OGM, although this was likely driven by OGM for negative cues.

The prospective relationship between OGM for negative cues and depressive symptoms has not been previously reported in studies of community samples (Crane et al., 2016; Gutenbrunner et al., 2017), but is consistent with one study of the adolescent offspring of depressed parents (Rawal and Rice, 2012). Effect sizes for the prospective association were smaller in ALSPAC (β = 0.074) compared to that observed in the study of Rawal and Rice (β = 0.12), which is one potential explanation as to why previous community studies with modest sample sizes have failed to find a prospective association. We found no evidence for gender or maternal depression status moderating this relationship. Results suggest that OGM may be a risk factor that precedes depression in the general population, not just in high-risk samples. If this is a causal relationship, then reducing OGM for negative material could have the potential to treat or prevent adolescent depression at a population level. Universal (or population level) preventative interventions for mental health disorders have received increased interest in recent years, but given the complex aetiology of such disorders it is necessary that interventions target multiple causal risk factors (Arango et al., 2018). Interventions that target cognitive processing (e.g. reducing OGM) may be one potential route to reducing depressive symptoms but OGM may also be a feature of a broader problem with overgeneralisation or adaptive emotion processing (Williams et al., 2007). Nonetheless, the prospective association between OGM and depressive symptoms is small, and interventions will likely need to target multiple routes to reduce depressive symptoms.

Results from this study contrast with those based on a previous analysis of ALSPAC by Crane et al. (2016). These differences may be due to the definition and valence of autobiographical memory used. Like Crane and colleagues, we found no prospective association between total specific memories and depression, but we did observe an association between OGM for negative cues and prospective depressive symptoms. Previous work based on ALSPAC, suggested that positive and negative responses on the AMT lie on the same continuum (Heron et al., 2012). Nevertheless, there is strong empirical evidence and theoretical grounding to suggest that adolescent depression may be associated with memories elicited by negative cues (Gotlib and Joormann, 2010; Rawal and Rice, 2012; Timbremont and Braet, 2004; Williams et al., 2007). This prospective relationship between OGM for negative cues was observed for both self- and parent-reported adolescent depressive symptoms.

Strengths of this study include use of a large, broadly representative sample (Boyd et al., 2013) and the prospective study design whereby the relationship between OGM and adolescent depressive symptoms was examined over a 3-year period. Limitations that merit consideration are attrition meaning that the analysed sample was more affluent, had more females and higher maternal education than the baseline sample (see Supplementary Material). Multiple imputation was used to reduce potential bias in complete case analyses but still relies on the assumption that data is missing at random which cannot be tested directly, meaning results here may differ slightly from the general UK population. Second, the use of the same informant to report on memory and depressive symptoms may introduce shared method bias, increasing the association between overgeneral memory and depression. Nevertheless, results replicated using parent-reported symptoms and, as a performance-based task, the AMT is potentially less affected by shared method variance. Third, self-reported depression (12.5 years) was not assessed at exactly the same point as the AMT (13 years). Thus, although OGM temporally precedes depression in prospective analyses, it is not clear whether the participants had depressive symptoms exactly when OGM was measured meaning unmeasured depression at 13 may have influenced results.

## Conclusion

Overgeneral autobiographical memory for negative cues was cross-sectionally and longitudinally associated with adolescent depressive symptoms in a large population-based sample. Memory biases for the personal past can precede the development of adolescent depression and may be a risk factor for later adolescent depressive symptoms.

## Contributors (CRediT authorship contribution statement)

Naomi Warne: Conceptualization, Formal analysis, Writing – original draft, Writing – review and editing. Xavier Caseras: Supervision, Writing – review and editing. France Rice: Conceptualization, Supervision, Writing - review & editing.

## Funding

NW was supported by a Medical Research Council studentship under MR/K501347/1. This work was supported by a seed corn grant from the MRC Centre for Neuropsychiatric Genetics and Genomics (FR) and MRC grant MR/R004609/1. The UK Medical Research Council and Wellcome (Grant ref: 102215/2/13/2) and the University of Bristol provide core support for ALSPAC. This publication is the work of the authors and Naomi Warne and Frances Rice will serve as guarantors for the contents of this paper. A comprehensive list of grants funding (PDF, 459 KB) is available on the ALSPAC website: http://www.bristol.ac.uk/alspac/external/documents/grant-acknowledgements.pdf. This research was specifically funded by Wellcome Trust and MRC (Grant ref: 92731). The funding sources had no role in the study design, analysis, decision to publish, or preparation of the manuscript.

## Declaration of Competing Interest

None.

## Appendix Supplementary materials

Image, application 1
