# Supplementary material for: The cross-sectional and longitudinal relationship between overgeneral autobiographical memory and adolescent depression in a UK population-based cohort
Source: J Affect Disord. 2020 Apr 1;266:621–5. doi: 10.1016/j.jad.2020.02.011 (PMC7097840; doi:10.1016/j.jad.2020.02.011)
Supplement: Supplementary file 1 [file mmc1.docx]

**Supplementary material**

**Autobiographical Memory Test**

Adolescents completed a written, minimal instruction version of the Autobiographical Memory Test (Crane et al., 2016; Heron et al., 2012; Williams and Broadbent, 1986). Participants were asked to write a real memory for 5 negative and 5 positive cue words presented in a pseudo-random order: happy, bored, relieved, hopeless, excited, failure, lonely, sad, lucky, relaxed. Responses were coded by ALSPAC staff (Crane et al., 2016; Heron et al., 2012) as either:

1. specific: a single event occurring on one day, at a particular time and place (e.g. ‘the day we first got my dog’);
2. extended: an event lasting two or more days (e.g. ‘when we went on holiday with my dog’);
3. categoric: repeated events within the same theme (e.g. ‘when I walk my dog’);
4. semantic associate: information pertaining to the cue word but not a memory (e.g. ‘my dog’); or
5. omissions: no responses and errors (e.g. statements about the future, statements about a lack of memory, or statements that were incomprehensible).

Inter-rater reliability was good to excellent between two initial raters (weighted κ = .82; unweighted κ = .78) and a third rater (weighted κ = .79; unweighted κ = .74) (Heron et al., 2012).

Measures used in the paper include specific responses and overgeneral responses (combined extended and categoric responses) in line with traditional coding systems (Williams et al., 2007; Williams and Broadbent, 1986). We used number of overgeneral responses to negative cues (potential range 0 to 5) as our primary variable. Secondary variables included total number of overgeneral responses (possible range 0 to 10), overgeneral responses to positive cues (possible range 0 to 5), total number of specific responses (possible range 0 to 10), specific responses to negative cues (possible range 0 to 5) and specific responses to positive cues (possible range 0 to 5). Specific measures are not the exact inverse of overgeneral measures as they do not take into account remaining coding categories (i.e. semantic associates and omissions).

**Missing data**

Attrition and missing data are often issues to consider in longitudinal studies (Gustavson et al., 2012; Sterne et al., 2009).

Total scores for the short Mood and Feelings Questionnaire (sMFQ) (Angold et al., 1995) were created by summing the scores and allowing for 20% missingness. This approach is recommended to account for small amounts of missingness in mental health measures (Goodman, 2001).

To account for potential bias caused by missing data in outcome and covariate data, additional analyses were conducted on an imputed dataset based on those with complete data on autobiographical memory and maternal depression status (n = 4111). Multiple imputation by chained equations was implemented using the ice command (Royston and White, 2011; White et al., 2011) in Stata version 13. Variables used in the imputation included those in the analysis models, variables that predicted missingness in the outcome and covariates (see Supplementary Tables 1 and 2), and total sMFQ scores associated with the outcomes (full list of variables available on request). Predictive mean matching was applied to continuous variables that were not normally distributed. One hundred imputed data sets were generated using ten cycles of regression switching.

Regression analyses were performed on the imputed datasets, combining estimates using Rubin’s rules (White et al., 2011). Using a two-stage calculation with a quadratic rule (von Hippel, 2018), the number of imputation was considered sufficient to ensure standard errors would not change considerably if imputed again (contemporaneous analysis imputations needed = 18; prospective analysis imputations needed = 98). FMI (fraction missing information) values were less than 1 and all Monte Carlo errors were less than 10% of the standard errors, indicating repeat imputation is likely to produce essentially the same results.

Key variable characteristics (means and percentages) were compared for complete cases and imputed datasets (Supplementary Table 3). Analyses performed on complete cases were repeated using imputed datasets and compared in Supplementary Table 4. As can be seen, imputed dataset results were largely consistent with complete case results, the only differences being maternal depression status (B (95% CI) = .317 (-.024, .658), p = .068) and gender (B (95% CI) = .217 (-.033, .467), p = .089) were no longer moderators of the AMSneg-contemporaneous depression relationship.

Supplementary Table 1. Indicators of incomplete data on depressive symptoms at baseline (12.5 years) and for contemporaneous analyses

(comparison amongst those with autobiographical memory and maternal depression status, n = 4111)

|  |  |  | **Depressive symptoms at baseline** | | | | **Depressive symptoms at baseline and covariates for contemporaneous analysis** | | | |
| --- | --- | --- | --- | --- | --- | --- | --- | --- | --- | --- |
| **Variable** | **Description** | **n** | **Complete data** | **Incomplete data** | **χ^2^** | **p** | **Complete data** | **Incomplete data** | **χ^2^** | **p** |
| Child sex | Male | 1854 | 1541 (83.12%) | 313 (16.88%) | 0.710 | 0.400 | 1410 (76.05%) | 444 (23.95%) | 0.847 | 0.357 |
|  | Female | 2257 | 1898 (84.09%) | 359 (15.91%) |  |  | 1744 (77.27%) | 513 (22.73%) |  |  |
| Tenure | Rent/other | 502 | 373 (74.30%) | 129 (25.70%) | 39.414 | **<.001** | 324 (64.54%) | 178 (35.46%) | 52.986 | **<.001** |
|  | Own/mortgage | 3434 | 2930 (85.32%) | 504 (14.68%) |  |  | 2717 (79.12%) | 717 (20.88%) |  |  |
| Overcrowding | <= 0.5 | 2089 | 1785 (85.45%) | 304 (14.55%) | 21.518 | **<.001** | 1672 (80.04%) | 417 (19.96%) | 34.521 | **<.001** |
|  | >0.5-0.75 | 1237 | 1038 (83.91%) | 199 (16.09%) |  |  | 937 (75.75%) | 300 (24.25%) |  |  |
|  | >0.75-1 | 541 | 425 (78.56%) | 116 (21.44%) |  |  | 377 (69.69%) | 164 (30.31%) |  |  |
|  | >1 | 141 | 107 (75.89%) | 34 (24.11%) |  |  | 96 (68.09%) | 45 (31.91%) |  |  |
| Parity | First born | 1980 | 1672 (84.44%) | 308 (15.56%) | 5.680 | 0.058 | 1552 (78.38%) | 428 (21.62%) | 9.312 | **0.010** |
|  | Second born | 1412 | 1192 (84.42%) | 220 (15.58%) |  |  | 1086 (76.91%) | 326 (23.09%) |  |  |
|  | Third or more born | 641 | 517 (80.66%) | 124 (19.34%) |  |  | 465 (72.54%) | 176 (27.46%) |  |  |
| Maternal education | Up to GCSE/  O level | 2168 | 1749 (80.67%) | 419 (19.33%) | 31.920 | **<.001** | 1568 (72.32%) | 600 (27.68%) | 55.406 | **<.001** |
|  | A level and above | 1902 | 1659 (87.22%) | 243 (12.78%) |  |  | 1563 (82.18%) | 339 (17.82%) |  |  |
| Maternal depression in pregnancy | No depression | 3730 | 3145 (84.32%) | 585 (15.68%) | 13.097 | **<.001** | 2915 (78.15%) | 815 (21.85%) | 45.759 | **<.001** |
|  | Depression | 375 | 289 (77.07%) | 86 (22.93%) |  |  | 235 (62.67%) | 140 (37.33%) |  |  |
| Maternal smoking in pregnancy | Did not smoke | 2793 | 2337 (83.67%) | 456 (16.33%) | 0.005 | 0.942 | 2160 (77.34%) | 633 (22.66%) | 2.099 | 0.147 |
|  | Smoked | 730 | 610 (83.56%) | 120 (16.44%) |  |  | 546 (74.79%) | 184 (25.21%) |  |  |
| Recurrent maternal depression status | Not depression | 3353 | 2849 (84.97%) | 504 (15.03%) | 22.999 | **<.001** | 2650 (79.03%) | 703 (20.97%) | 54.460 | **<.001** |
|  | Depression | 758 | 590 (77.84%) | 168 (22.16%) |  |  | 504 (66.49%) | 254 (33.51%) |  |  |
| Economic disadvantage | No | 2923 | 2502 (85.60%) | 421 (14.40%) | 16.771 | **<.001** | 2332 (79.78%) | 591 (20.22%) | 40.998 | **<.001** |
|  | Yes | 582 | 459 (78.87%) | 123 (21.13%) |  |  | 394 (67.70%) | 188 (32.30%) |  |  |
|  |  |  | **Depressive symptoms at baseline** | | | | **Depressive symptoms at baseline and covariates for contemporaneous analysis** | | | |
| **Variable** |  | **n** | **OR** | **95% CI** | | **p** | **OR** | **95% CI** | | **p** |
| Birthweight (kg) | | 4056 | 1.043 | 0.887, 1.226 | | 0.614 | 0.993 | 0.862, 1.144 | | 0.926 |
| Maternal age at birth | | 4111 | 0.963 | 0.945, 0.981 | | **<.001** | 0.952 | 0.937, 0.968 | | **<.001** |
| Child age at AMT | | 4111 | 1.045 | 0.982, 1.113 | | 0.167 | 1.051 | 0.993, 1.111 | | 0.085 |
| Child IQ |  | 3434 | 0.988 | 0.981, 0.996 | | **0.003** | 0.988 | 0.981, 0.996 | | **0.003** |
| Age 10 child reported depression (fddp) | | 3514 | 1.020 | 0.984, 1.057 | | 0.281 | 1.025 | 0.998, 1.053 | | 0.067 |
| Age 13.5 child reported depression (fg) | | 3210 | 1.018 | 0.978, 1.060 | | 0.384 | 1.012 | 0.987, 1.037 | | 0.360 |
| Age 9 parent reported depression (ku) | | 3888 | 1.003 | 0.975, 1.031 | | 0.831 | 1.016 | 0.992, 1.041 | | 0.181 |
| Age 11 parent reported depression (kw) | | 3813 | 1.027 | 0.999, 1.055 | | 0.056 | 1.027 | 1.002, 1.051 | | **0.031** |
| Age 13 parent reported depression (ta) | | 3856 | 1.020 | 0.995, 1.046 | | 0.123 | 1.027 | 1.004, 1.049 | | **0.019** |
| Age 16 parent reported depression (tc) | | 3289 | 1.005 | 0.977, 1.035 | | 0.722 | 1.019 | 0.995, 1.044 | | 0.117 |

Supplementary Table 2. Indicators of incomplete data on depressive symptoms at follow up (16 years) and for prospective analyses

(comparison amongst those with autobiographical memory and maternal depression status, n = 4111)

|  |  |  | **Depressive symptoms at follow up** | | | | **Depressive symptoms at follow up and covariates for prospective analysis** | | | |
| --- | --- | --- | --- | --- | --- | --- | --- | --- | --- | --- |
| **Variable** | **Description** | **n** | **Complete data** | **Incomplete data** | **χ^2^** | **p** | **Complete data** | **Incomplete data** | **χ^2^** | **p** |
| Child sex | Male | 1854 | 1188 (64.08%) | 666 (35.92%) | 78.704 | **<.001** | 952 (51.35%) | 902 (48.65%) | 44.675 | **<.001** |
|  | Female | 2257 | 1731 (76.69%) | 526 (23.31%) |  |  | 1393 (61.72%) | 864 (38.28%) |  |  |
| Tenure | Rent/other | 502 | 296 (58.96%) | 206 (41.04%) | 41.339 | **<.001** | 212 (42.23%) | 290 (57.77%) | 54.855 | **<.001** |
|  | Own/mortgage | 3434 | 2503 (72.89%) | 931 (27.11%) |  |  | 2051 (59.73%) | 1383 (40.27%) |  |  |
| Overcrowding | <= 0.5 | 2089 | 1580 (75.63%) | 509 (24.37%) | 51.797 | **<.001** | 1306 (62.52%) | 783 (37.48%) | 59.635 | **<.001** |
|  | >0.5-0.75 | 1237 | 851 (68.80%) | 386 (31.20%) |  |  | 667 (53.92%) | 570 (46.08%) |  |  |
|  | >0.75-1 | 541 | 338 (62.48%) | 203 (37.52%) |  |  | 258 (47.69%) | 283 (52.31%) |  |  |
|  | >1 | 141 | 85 (60.28%) | 56 (39.72%) |  |  | 62 (43.97%) | 79 (56.03%) |  |  |
| Parity | First born | 1980 | 1441 (72.78%) | 539 (27.22%) | 7.745 | **0.021** | 1181 (59.65%) | 799 (40.35%) | 11.846 | **0.003** |
|  | Second born | 1412 | 1002 (70.96%) | 410 (29.04%) |  |  | 793 (56.16%) | 619 (43.84%) |  |  |
|  | Third or more born | 641 | 430 (67.08%) | 211 (32.92%) |  |  | 335 (52.26%) | 306 (47.74%) |  |  |
| Maternal education | Up to GCSE/  O level | 2168 | 1410 (65.04%) | 758 (34.96%) | 88.256 | **<.001** | 1080 (49.82%) | 1088 (50.18%) | 108.286 | **<.001** |
|  | A level and above | 1902 | 1491 (78.39%) | 411 (21.61%) |  |  | 1255 (65.98%) | 647 (34.02%) |  |  |
| Maternal depression in pregnancy | No depression | 3730 | 2694 (72.23%) | 1036 (27.77%) | 25.869 | **<.001** | 2188 (58.66%) | 1542 (41.34%) | 40.483 | **<.001** |
|  | Depression | 375 | 224 (59.73%) | 151 (40.27%) |  |  | 156 (41.60%) | 219 (58.40%) |  |  |
| Maternal smoking in pregnancy | Did not smoke | 2793 | 2025 (72.50%) | 768 (27.50%) | 8.617 | **0.003** | 1629 (58.32%) | 1164 (41.68%) | 7.015 | **0.008** |
|  | Smoked | 730 | 489 (66.99%) | 241 (33.01%) |  |  | 386 (52.88%) | 344 (47.12%) |  |  |
| Recurrent maternal depression status | Not depression | 3353 | 2449 (73.04%) | 904 (26.96%) | 36.559 | **<.001** | 2004 (59.77%) | 1349 (40.23%) | 55.119 | **<.001** |
|  | Depression | 758 | 470 (62.01%) | 288 (37.99%) |  |  | 341 (44.99%) | 417 (55.01%) |  |  |
| Economic disadvantage | No | 2923 | 2137 (73.11%) | 786 (26.89%) | 17.997 | **<.001** | 1758 (60.14%) | 1165 (39.86%) | 31.390 | **<.001** |
|  | Yes | 582 | 375 (64.43%) | 207 (35.57%) |  |  | 277 (47.59%) | 305 (52.41%) |  |  |
|  | |  | **Depressive symptoms at follow up** | | | | **Depressive symptoms at follow up and covariates for prospective analysis** | | | |
| **Variable** | | **n** | **OR** | **95% CI** | | **p** | **OR** | **95% CI** | | **p** |
| Birthweight (kg) |  | 4056 | 1.153 | 1.010, 1.317 | | **0.035** | 1.087 | 0.964, 1.227 | | 0.174 |
| Maternal age at birth |  | 4111 | 0.950 | 0.936, 0.965 | | **<.001** | 0.948 | 0.935, 0.961 | | **<.001** |
| Child age at AMT |  | 4111 | 1.264 | 1.190, 1.343 | | **<.001** | 1.232 | 1.156, 1.313 | | **<.001** |
| Child IQ |  | 3434 | 0.979 | 0.975, 0.984 | | **<.001** | 0.981 | 0.976, 0.985 | | **<.001** |
| Age 10 child reported depression (fddp) | | 3514 | 1.002 | 0.981, 1.024 | | 0.867 | 1.004 | 0.984, 1.024 | | 0.718 |
| Age 12.5 child reported depression (ff) | | 3439 | 0.999 | 0.979, 1.019 | | 0.887 | 1.005 | 0.986, 1.024 | | 0.617 |
| Age 13.5 child reported depression (fg) | | 3210 | 1.012 | 0.994, 1.030 | | 0.184 | 1.015 | 0.998, 1.032 | | 0.081 |
| Age 9 parent reported depression (ku) | | 3888 | 1.038 | 1.016, 1.061 | | **0.001** | 1.029 | 1.008, 1.050 | | **0.006** |
| Age 11 parent reported depression (kw) | | 3813 | 1.047 | 1.024, 1.070 | | **<.001** | 1.039 | 1.017, 1.061 | | **<.001** |
| Age 13 parent reported depression (ta) | | 3856 | 1.047 | 1.026, 1.068 | | **<.001** | 1.044 | 1.024, 1.064 | | **<.001** |
| Age 16 parent reported depression (tc) | | 3289 | 1.026 | 1.002, 1.051 | | **0.036** | 1.027 | 1.006, 1.049 | | **0.011** |

Supplementary Table 3. Observed (complete case) versus imputed descriptive analysis on key variables

|  | |  | **Complete case**  **% / mean (SD)** | **Imputed**  **% / mean (SD)** |
| --- | --- | --- | --- | --- |
| Economic disadvantage | |  | 16.60% | 17.02% |
| IQ | |  | 107.235 (15.820) | 106.757 (15.914) |
| Baseline depressive symptoms (age 12.5) | |  | 3.965 (3.791) | 3.988 (3.820) |
| Follow up depressive symptoms (age 16) | |  | 5.789 (5.547) | 5.797 (5.578) |
| Tenure (own/mortgage) | |  | 87.25% | 86.62% |
| Maternal education (A level and above) | |  | 46.73% | 46.55% |
| Maternal smoking in pregnancy | |  | 20.72% | 20.82% |
| Parity | | |  |  |
|  | First born | | 49.09% | 49.15% |
|  | Second born | | 35.01% | 34.98% |
|  | Third+ born | | 15.89% | 15.87% |
| Crowding | | |  |  |
|  | <=0.5 | | 52.12% | 51.94% |
|  | >0.5-0.75 | | 30.86% | 30.89% |
|  | >0.75-1 | | 13.50% | 13.58% |
|  | >1 | | 3.52% | 3.59% |
| Maternal depression in pregnancy | |  | 9.14% | 9.20% |
| Parent-reported depressive symptoms (age 13) (ta) | |  | 2.336 (3.328) | 2.382 (3.372) |
| Parent-reported depressive symptoms (age 16) (tc) | |  | 2.111 (3.386) | 2.170 (3.455) |
| Parent-reported depressive symptoms (age 9) (ku) | |  | 2.442 (3.107) | 2.484 (3.140) |
| Parent-reported depressive symptoms (age 11) (kw) | |  | 2.223 (3.070) | 2.257 (3.100) |
| Child-reported depressive symptoms (age 10) (fddp) | |  | 3.921 (3.465) | 3.958 (3.491) |
| Child-reported depressive symptoms (age 13.5) (fg) | |  | 4.909 (4.447) | 4.944 (4.478) |

Supplementary Table 4. Comparison of regression models for complete case and imputed data

| **Analysis** | | **Contemporaneous depressive symptoms** | | | | **Prospective depressive symptoms** | | | |
| --- | --- | --- | --- | --- | --- | --- | --- | --- | --- |
|  |  | **Complete case** | | **Imputed data** | | **Complete case** | | **Imputed data** | |
|  |  | **B (95% CI)** | **p** | **B (95% CI)** | **p** | **B (95% CI)** | **p** | **B (95% CI)** | **p** |
| **OGMneg main analysis** | |  |  |  |  |  |  |  |  |
|  | OGMneg main effect | **.406 (.275, .537)** | **<.001** | **.416 (.290, .542)** | **<.001** | **.410 (.202, .617)** | **<.001** | **.396 (.196, .596)** | **<.001** |
|  | OGMneg x maternal depression status | .159 (-.202, .519) | .388 | .096 (-.229, .422) | .561 | -.080 (-.675, 514) | **.791** | -.112 (-.611, .387) | .660 |
|  | OGMneg x gender | .093 (-.169, .354) | .488 | .083 (-.170, .335) | .521 | -.180 (-.595, .235) | .395 | .103 (-.271, .478) | .588 |
| **Testing specificity of OGMneg relationships** | | |  |  |  |  |  |  |  |
|  | OGMtotal main effect | **.264 (.130, .398)** | **<.001** | **.265 (.137, .392)** | **<.001** | **.344 (.133, .556)** | **.001** | **.346 (.141, .551)** | **.001** |
|  | OGMtotal x maternal depression status | -.059 (-.430, .312) | .755 | <.001 (-.330, .330) | 1 | .060 (-.554, .674) | .847 | .046 (-.454, .546) | .857 |
|  | OGMtotal x gender | .095 (-.172, .362) | .485 | .056 (-.198, .310) | .665 | -.111 (-.532, .311) | .607 | .032 (-.343, .407) | .868 |
|  | OGMpos main effect | .031 (-.103, .166) | .649 | .031 (-.098, .160) | .636 | .168 (-.044, .381) | .120 | .198 (-.001, .397) | .052 |
|  | OGMpos x maternal depression status | -.276 (-.643, .091) | .140 | -.111 (-.440, .219) | .510 | .169 (-.440, .779) | .586 | .198 (-.289, .685) | .425 |
|  | OGMpos x gender | .040 (-.227, .308) | .767 | -.024 (-.277, .229) | .853 | -.023 (-.445, .400) | .917 | -.075 (-.441, .290) | .686 |
|  | AMStotal main effect | **.179 (.045, .314)** | **.009** | **.180 (.051, .309)** | **.006** | .053 (-.157, .263) | .618 | .027 (-.167, .222) | .782 |
|  | AMStotal x maternal depression status | **.579 (.222, .937)** | **.002** | **.426 (.093, .760)** | **.012** | -.319 (-.905, .267) | .286 | -.193 (-.695, .310) | .452 |
|  | *Follow up: AMStotal in no maternal depression* | *.091 (-.054, .236)* | *.219* | *.103 (-.036, .242)* | *.145* |  |  |  |  |
|  | *Follow up: AMStotal in maternal depression* | ***.673 (.318, 1.028)*** | ***<.001*** | ***.523 (.189, .857)*** | ***.002*** |  |  |  |  |
|  | AMStotal x gender | .164 (-.103, .431) | .229 | .189 (-.061, .439) | .139 | .107 (-.314, .529) | .617 | .084 (-.274, .441) | .646 |
|  | AMSneg main effect | **.354 (.222, .486)** | **< .001** | **.349 (.220, .478)** | **<.001** | .079 (-.125, .283) | .446 | .054 (-.136, .244) | .575 |
|  | AMSneg x maternal depression status | **.363 (.003, .723)** | **.048** | .317 (-.024, .658) | .068 | -.322 (-.897, .254) | .273 | -.138 (-.649, .373) | .595 |
|  | *Follow up: AMSneg in no maternal depression* | ***.302 (.161, .444)*** | ***<.001*** |  |  |  |  |  |  |
|  | *Follow up: AMSneg in maternal depression* | ***.659 (.301, 1.017)*** | ***<.001*** |  |  |  |  |  |  |
|  | AMSneg x gender | **.269 (.005, .533)** | **.046** | .225 (-.025, .474) | .078 | .190 (-.224, .604) | .368 | .046 (-.309, .400) | .800 |
|  | *Follow up: AMSneg in males* | ***.225 (.058, .391)*** | ***.008*** |  |  |  |  |  |  |
|  | *Follow up: AMSneg in females* | ***.461 (.299, .624)*** | ***<.001*** |  |  |  |  |  |  |
|  | AMSpos main effect | -.028 (-.162, .107) | .685 | -.017 (-.144, .111) | .799 | .015 (-.198, .227) | .893 | -.003 (-.198, .191) | .974 |
|  | AMSpos x maternal depression status | **.663 (.308, 1.019)** | **<.001** | **.444 (.117, .771)** | **.008** | -.241 (-.833, .351) | .425 | -.197 (-.688, .293) | .429 |
|  | *Follow up: AMSpos in no maternal depression* | *-.132 (-.277, .014)* | *.076* | *-.098 (-.236, .040)* | *.164* |  |  |  |  |
|  | *Follow up: AMSpos in maternal depression* | ***.531 (.180, .882)*** | ***.003*** | ***.337 (.014, .660)*** | ***.041*** |  |  |  |  |
|  | AMSpos x gender | .002 (-.266, .269) | .990 | .087 (-.164, .338) | .496 | <.001 (-.426, .425) | 1 | .095 (-.267, .458) | .606 |
| **Sensitivity analyses** | |  |  |  |  |  |  |  |  |
| *Parent reported depressive symptoms* | | |  |  |  |  |  |  |  |
|  | OGMneg main effect | **.262 (.151, .372)** | **<.001** | **.281 (.176, .385)** | **<.001** | **.213 (.100, .325)** | **<.001** | **.202 (.091, .312)** | **<.001** |
|  | OGMneg x maternal depression status | .073 (-.227, .373) | .634 | .028 (-.246. .303) | .840 | .053 (-.262, .367) | .743 | -.052 (-.330, .226) | .711 |
|  | OGMneg x gender | .022 (-.199, .243) | .845 | .071 (-.140, .281) | .512 | .093 (-.130, .316) | .412 | .194 (-.014, .402) | .068 |
| *Adjusting for SES* | |  |  |  |  |  |  |  |  |
|  | OGMneg main effect | **.398 (.258, .539)** | **<.001** | **.416 (.290, .541)** | **<.001** | **.431 (.205, .657)** | **<.001** | **.396 (.196, .596)** | **<.001** |
|  | OGMneg x maternal depression status | .147 (-.255, .548) | .474 | .088 (-.237, .413) | .596 | -.185 (-.841, .471) | .579 | -.114 (-.613, .385) | .654 |
|  | OGMneg x gender | .091 (-.189, .372) | .523 | .096 (-.157, .348) | .458 | -.193 (-.644, .258) | .401 | .096 (-.278, .471) | .614 |

AMSneg – Specific autobiographical memories to negative cues; AMSpos – Specific autobiographical memories to positive cues; AMStotal – Total specific autobiographical memories; OGMneg – Overgeneral autobiographical memories to negative cues; OGMpos - Overgeneral autobiographical memories to positive cues; OGMtotal – Total overgeneral autobiographical memories. Results significant at p < .05 are indicated in bold.

**References**

Angold, A., Costello, E.J., Messer, S.C., Pickles, A., Winder, F., Silver, D., 1995. The development of a short questionnaire for use in epidemiological studies of depression in children and adolescents. Int. J. Methods Psychiatr. Res. 5, 237–249.

Crane, C., Heron, J., Gunnell, D., Lewis, G., Evans, J., Williams, J.M.G., 2016. Adolescent over-general memory, life events and mental health outcomes: Findings from a UK cohort study. Memory 24, 348–363.

Goodman, R., 2001. Psychometric properties of the strengths and difficulties questionnaire. J. Am. Acad. Child Adolesc. Psychiatry 40, 1337–1345.

Gustavson, K., von Soest, T., Karevold, E., Røysamb, E., 2012. Attrition and generalizability in longitudinal studies: findings from a 15-year population-based study and a Monte Carlo simulation study. BMC Public Health 12, 918. https://doi.org/10.1186/1471-2458-12-918

Heron, J., Crane, C., Gunnell, D., Lewis, G., Evans, J., Williams, J.M., 2012. 40,000 memories in young teenagers: Psychometric properties of the Autobiographical Memory Test in a UK cohort study. Memory 20, 300–320.

Royston, P., White, I.R., 2011. Multiple Imputation by Chained Equations (MICE): Implementation in Stata. J. Stat. Softw. 45, 1–20. https://doi.org/10.1002/wics.10

Sterne, J.A.C., White, I.R., Carlin, J.B., Spratt, M., Royston, P., Kenward, M.G., Wood, A.M., Carpenter, J.R., 2009. Multiple imputation for missing data in epidemiological and clinical research: potential and pitfalls. BMJ 338, b2393.

von Hippel, P.T., 2018. How Many Imputations Do You Need? A Two-stage Calculation Using a Quadratic Rule. Sociol. Methods Res. 004912411774730. https://doi.org/10.1177/0049124117747303

White, I.R., Royston, P., Wood, A.M., 2011. Multiple imputation using chained equations: Issues and guidance for practice. Stat. Med. 30, 377–399. https://doi.org/10.1002/sim.4067

Williams, J.M.G., Barnhofer, T., Crane, C., Hermans, D., Raes, F., Watkins, E., Dalgleish, T., 2007. Autobiographical memory specificity and emotional disorder. Psychol. Bull. 133, 122–148.

Williams, J.M.G., Broadbent, K., 1986. Autobiographical memory in suicide attempters. J. Abnorm. Psychol. 95, 144–149.
